# Direct pathway cloning of the sodorifen biosynthetic gene cluster and recombinant generation of its product in *E. coli*

**DOI:** 10.1186/s12934-019-1080-6

**Published:** 2019-02-07

**Authors:** Elke R. Duell, Paul M. D’Agostino, Nicole Shapiro, Tanja Woyke, Thilo M. Fuchs, Tobias A. M. Gulder

**Affiliations:** 1 Biosystems Chemistry, Department of Chemistry and Center for Integrated Protein Science Munich (CIPSM), Technical University of Munich, Lichtenbergstraße 4, 85748 Garching, Germany; 2 Department of Energy, Joint Genome Institute, 2800 Mitchell Drive, Walnut Creek, CA 94598 USA; 3 ZIEL Institute for Food & Health, Lehrstuhl für Mikrobielle Ökologie, Department biowissenschaftliche Grundlagen, Technical University of Munich, Munich, Germany; 4 Friedrich-Loeffler-Institut, Institut für Molekulare Pathogenese, Jena, Germany; 5 Chair of Technical Biochemistry, Technische Universität Dresden, Bergstraße 66, 01602 Dresden, Germany

**Keywords:** Sodorifen, *Serratia plymuthica*, Terpenes, Genome mining, DiPaC, Heterologous expression

## Abstract

**Background:**

*Serratia plymuthica* WS3236 was selected for whole genome sequencing based on preliminary genetic and chemical screening indicating the presence of multiple natural product pathways. This led to the identification of a putative sodorifen biosynthetic gene cluster (BGC). The natural product sodorifen is a volatile organic compound (VOC) with an unusual polymethylated hydrocarbon bicyclic structure (C_16_H_26_) produced by selected strains of *S. plymuthica.* The BGC encoding sodorifen consists of four genes, two of which (*sodA*, *sodB*) are homologs of genes encoding enzymes of the non-mevalonate pathway and are thought to enhance the amounts of available farnesyl pyrophosphate (FPP), the precursor of sodorifen. Proceeding from FPP, only two enzymes are necessary to produce sodorifen: an *S*-adenosyl methionine dependent methyltransferase (SodC) with additional cyclisation activity and a terpene-cyclase (SodD). Previous analysis of *S. plymuthica* found sodorifen production titers are generally low and vary significantly among different producer strains. This precludes studies on the still elusive biological function of this structurally and biosynthetically fascinating bacterial terpene.

**Results:**

Sequencing and mining of the *S. plymuthica* WS3236 genome revealed the presence of 38 BGCs according to antiSMASH analysis, including a putative sodorifen BGC. Further genome mining for sodorifen and sodorifen-like BGCs throughout bacteria was performed using SodC and SodD as queries and identified a total of 28 *sod*-like gene clusters. Using direct pathway cloning (DiPaC) we intercepted the 4.6 kb candidate sodorifen BGC from *S. plymuthica* WS3236 (*sodA*–*D*) and transformed it into *Escherichia coli* BL21. Heterologous expression under the control of the tetracycline inducible P*tet*_*O*_ promoter firmly linked this BGC to sodorifen production. By utilizing this newly established expression system, we increased the production yields by approximately 26-fold when compared to the native producer. In addition, sodorifen was easily isolated in high purity by simple head-space sampling.

**Conclusions:**

Genome mining of all available genomes within the NCBI and JGI IMG databases led to the identification of a wealth of *sod*-like pathways which may be responsible for producing a range of structurally unknown sodorifen analogs. Introduction of the *S. plymuthica* WS3236 sodorifen BGC into the fast-growing heterologous expression host *E. coli* with a very low VOC background led to a significant increase in both sodorifen product yield and purity compared to the native producer. By providing a reliable, high-level production system, this study sets the stage for future investigations of the biological role and function of sodorifen and for functionally unlocking the bioinformatically identified putative *sod*-like pathways.

**Electronic supplementary material:**

The online version of this article (10.1186/s12934-019-1080-6) contains supplementary material, which is available to authorized users.

## Background

**Fig. 1:**
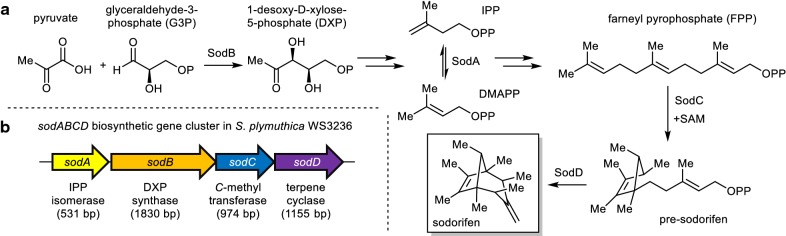
**a** Biosynthesis of the polymethylated hydrocarbon sodorifen (C_16_H_26_) from MEP-pathway-derived farnesyl pyrophosphat (FPP) via the intermediate pre-sodorifen [13]. **b** Sodorifen BGC from *S. plymuthica* WS3236 harboring an isopentenyl-diphosphate delta-isomerase *sodA*, a 1-deoxy-d-xylulose-5-phosphate synthase *sodB*, a SAM-dependent C-methyltransferase *sodC* and a terpene cyclase *sodD*

Volatile organic compounds (VOCs) are lipophilic small molecules (< 300 Da) that are characterized by their high vapor pressures and low boiling points. They play important biological roles, e.g., as signaling molecules by enabling communication over long-distances and are able to facilitate cross-kingdom interactions [1–3]. Thus, understanding their ecological functions is important, for instance when looking deeper into microbial ecology or plant-rhizosphere interactions. The rhizobacterium *Serratia plymuthica* 4Rx13, for example, is emitting over 100 different VOCs which have been shown to inhibit plant and fungal growth [4–9]. The VOC profile of *S. plymuthica* 4Rx13 contains sodorifen as one major metabolite, a polymethylated hydrocarbon with an unusual bicyclo[3.2.1]octadiene skeleton (Fig. 1a) [10].

Recently, a 4.6 kb sodorifen (*sod*) BGC cluster harboring four individual genes was found to be responsible for the sodorifen production in *S. plymuthica* 4Rx13 (Fig. 1b) [11]. The first two genes, an isopentenyl-diphosphate delta-isomerase (IPP isomerase, *sodA*) and a 1-deoxy-d-xylulose-5-phosphate synthase (DXP synthase, *sodB*) are homologs of genes found in the non-mevalonate pathway (MEP-pathway) and may supply additional farnesyl-pyrophosphat (FPP), the precursor of sodorifen. Notably, knock-out-mutants showed that SodB is not crucial for sodorifen emission, most likely due to the fact that one of the house-keeping enzymes of the MEP-pathway provides sufficient precursor flux [11]. Expression of *sodCD* in *Escherichia coli* additionally harboring the plasmid pMEV with three MEP-pathway associated enzymes led to the production of minute amounts of sodorifen and its isomers [12]. Recent investigations in vivo and in vitro demonstrated that the SAM-dependent *C*-methyltransferase SodC is not only responsible for the addition of a methyl group to FPP, but also exhibits a surprising cyclisation activity leading to the phosphorylated version of pre-sodorifen, which is then cyclized by the terpene cyclase SodD to yield the final bicyclic compound sodorifen (Fig. 1a) [13].

Interestingly, sodorifen is produced by multiple members of the rhizobacterium family of *S. plymuthica* [10] suggesting an important ecological role of this specialized metabolite. Most *S. plymuthica* strains investigated so far emit a complex mixture of different VOCs mainly at very low concentrations [4], greatly hampering the efficient production for functional screening of individual compounds such as sodorifen. Sodorifen production can be triggered to some extend by co-cultivating *S. plymuthica* with the fungal pathogen *Fusarium culmorum* [12] and is furthermore regulated by the carbon catabolite repression system [14]. Different isolates of *S. plymuthica* exhibit surprisingly variable amounts of sodorifen emission, ranging from < 0.1 to 50% of the total VOC spectrum under identical fermentation conditions [14]. Given the rather low titers of sodorifen in all natural producers, its inherent volatility and the observed complexity of VOC mixtures obtained by fermentation of *S. plymuthica*, studies on the functional role of this intriguing metabolite have so far been impeded. Optimized biotechnological production of sodorifen in a suitable recombinant host with low VOC background has the potential to solve this apparent supply problem thus enabling in-depth functional studies. Such an approach requires the cloning and successful heterologous, functional expression of the encoding biosynthetic gene cluster. Biosynthetic gene clusters can be captured using several in vivo methods based on recombineering, such as liner-linear homologous recombineering (LLHR) [15] or linear-circular-homologous recombineering (LCHR) [16], exonuclease combined recombination (ExoCET) [17] and Cas9-assisted targeting of chromosome segments (CATCH) [18], all utilizing the Rec/ET system [19], or using the natural recombination capability of *Saccharomyces cerevisiae* for transformation-associated recombination (TAR) cloning [20–22]. In addition to this, there are PCR based in vitro BGC cloning techniques such as circular polymerase extension cloning (CPEC) [23], assembly of fragment ends after PCR (AFEAP) [24] and direct pathway cloning (DiPaC) [25, 26]. DiPaC is characterized by long-amplicon PCR combined with homologous nucleotide overhangs which allows for in vitro DNA assembly via Gibson assembly or sequence- and ligation-independent cloning (SLIC) of the BGC directly into the expression vector of choice. This enables the rapid, efficient and cheap capturing and expression of BGCs of interest in heterologous hosts such as *E. coli* or *Streptomyces* spp. [25, 26]. Within this study we present the application of DiPaC to functionally link a putative sodorifen biosynthetic gene cluster to sodorifen production in a recombinant host system and the optimization of the fermentative strategy to almost exclusive, high-level sodorifen production.

## Results

*Serratia plymuthica* WS3236 was selected for WGS after preliminary genetic and chemical screening (data not shown) that pointed to the presence of natural product pathways. The resulting genome has a GC content of 55.93% and encodes a total of 5107 genes, of which 4915 (96.24%) are protein encoding genes. The genome sequence has been deposited at GenBank SRA (BioProject ID: PRJNA442736) and IMG/MER (Accession: 2773857786) databases. To disclose the secondary metabolite potential of *S. plymuthica* WS3236, an in silico approach utilizing antiSMASH [27] and ClusterFinder [28] algorithms was performed. A total of 38 BGCs were identified (Additional file 1: Table S1), of which two displayed high homology to known BGCs encoding zeamine [29] (MIBiG BGC-ID: BGC0001056_c1) and sodorifen [11, 13] (MIBiG BGC-ID: BGC0001361_c1). A further seven did not show similarity to characterized pathways but belonged to well-known natural product families including non-ribosomal peptide synthase (NRPS; 4 clusters), arylpolyene-siderophore (1 cluster), polyketide synthetase/NRPS (PKS/NRPS; 1 cluster) and thiopeptide (1 cluster) biosynthesis (Additional file 1: Table S1); the remaining 29 pathways were predicted by ClusterFinder and either belonged to fatty acid, saccharide or putative pathways. Within this work, we decided to further investigate the candidate sodorifen BGC, to (1) firmly validate its small molecule product and (2) establish a reliable high-level recombinant system for sodorifen production that will facilitate future investigations into its biological functions.

**Fig. 2:**
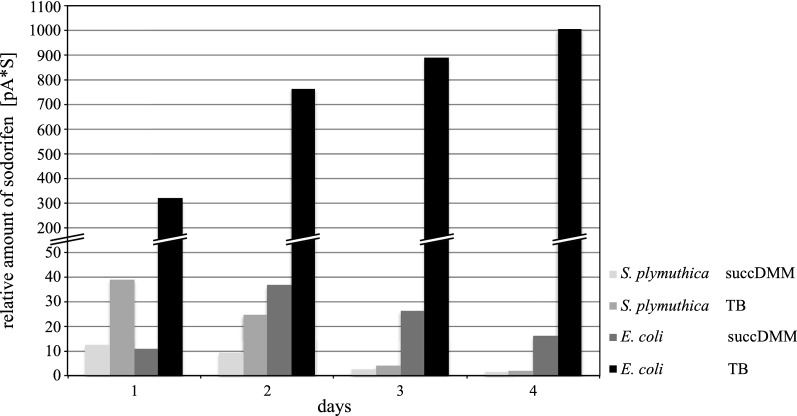
Quantitative comparison of sodorifen amounts produced by *S. plymuthica* WS3236 and *E. coli* cultivated in succDMM and TB media over the course of 4 days. Yields were calculated based on peak areas pA*s measured with analytical GC. 26-fold higher sodorifen production in *E. coli* (TB, 4th day) can be observed compared to the best conditions tested for *S. plymuthica* (TB, 1st day). *S. plymuthica* shows a constant decrease of sodorifen production during the time course whereas for *E. coli* in TB a constant increase is observed and in succDMM a maximum is reached on the 2nd day

To functionally link the *sod* candidate BGC to sodorifen production, *S. plymuthica* WS3236 was cultivated either in TB or succDMM, the latter being reported as the optimal sodorifen production medium for *S. plymuthica* [13]. Sodorifen yields were highest after day 1 and constantly decreased after this time point (Fig. 2). Interestingly, we found that cultivation in TB resulted in slightly higher sodorifen emission, which might solely be a consequence of higher growth rates and cell densities compared to the minimal succDMM medium. As neither TB nor succDMM contain glucose, the carbon catabolite repression system regulating the *sod* BGC, as described by Magnus et al. [14], does not come into effect. Most importantly, all production experiments with the natural producer *S. plymuthica* WS3236 only resulted in small amounts of sodorifen relative to other VOCs produced. As purification of highly volatile organic compounds is generally difficult, we set out to construct a heterologous production system that reliably gives access to larger amounts of sodorifen, most importantly with high purity by simple head-space sampling.

**Fig. 3:**
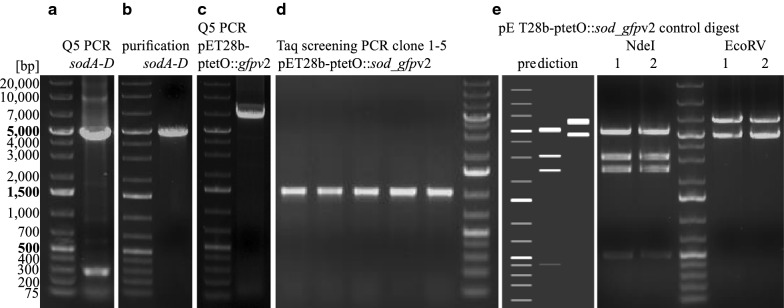
DiPaC of the *sod* BGC into pET28b-ptetO::*sod_gfp*v2. **a** PCR amplification of the 4.6 kb *sodA*–*D* fragment with Q5 polymerase and primers containing homology sequences for SLIC into the backbone, **b** *sodA–D* fragment after gel purification, **c** PCR amplification to construct a linear 6.6 kb backbone of pET28b-ptetO::*gfp*v2, **d** positive screening PCR of five clones after transformation of the SLIC-joined pET28b-ptetO::*sod_gfp*v2 plasmid and **e** control restriction digest of two clones with *Nde*I or *Eco*RV in comparison to the predicted (Snapgene) pattern

For the interception of the *sod* BGC the DiPaC strategy was applied. An expression plasmid containing the tetracycline inducible promoter P*tet*_*O*_ was chosen for heterologous expression as the use of stronger promoters such as T7 has previously been shown to frequently hamper secondary metabolite production [30]. Therefore the 4.6 kb *sod* BGC was PCR amplified in one piece using primers equipped with homologous overhangs for the vector backbone pET28b-ptetO::*gfp*v2 (see Fig. 3a, b). This vector carries a copy of the *gfp* gene downstream of the multiple cloning site thus enabling the validation of transcription/translation of the complete construct by simple fluorescence detection of GFP. After linearizing the vector backbone by PCR (see Fig. 3c), the two fragments where joined using SLIC. Successful integration of the *sod* BGC into pET28b-ptetO::*gfp*v2 was verified by colony screening PCR, analytical restriction digest and sequencing of the integration site of the resulting plasmid pET28b-ptetO::*sod_gfp*v2 (see Fig. 3 and Additional file 1: Figure S2).

Full transcription/translation of the *sod* BGC under the control of P*tet*_*O*_ was confirmed by detecting green fluorescence in the presence of tetracycline, but not in the controls (see Additional file 1: Figure S3). SDS gel analysis of the whole cell protein of *S. plymuthica* WS3236, *E. coli* BL21 as well as induced versus uninduced *E. coli* carrying plasmid pET28b-ptetO::*sod_gfp*v2 did not reveal any differences in the protein expression pattern (see Additional file 1: Figure S4). This clearly underlines the versatility of the applied fluorescence screening using the GFP reporter protein downstream of the *sod* pathway to prove successful transcription/translation of the entire construct. GC–MS analysis of all VOCs emitted into the headspace of the heterologous fermentation in all three tested media revealed the accumulation of a molecule with a molecular mass of *m/z* 218 [M^+^] which clearly showed the typical mass spectrum of sodorifen [10] (see Additional file 1: Figure S5). In addition, low amounts of sodorifen isomers known to also be produced by *S. plymuthica* were likewise observed [13]. As no potential sodorifen pathway intermediates were detected, apparently all terpenoid precursors available to the sodorifen biosynthetic system were fully converted to the end product by SodC and SodD.

Optimization of the heterologous production of sodorifen was tested in three different media: LB, TB and succDMM. The amounts of emitted VOC products were compared to those of *S. plymuthica* WS3236 under the same growth conditions. Using succDMM as cultivation medium for *E. coli* BL21 pET28b-ptetO::*sod_gfp*v2, the yield of emitted sodorifen reached a maximum on day 2 and was comparable to the production of *S. plymuthica* WS3236 grown in TB (cf. Fig. 2). Performing the heterologous expression in LB medium showed a maximal production on day 2, and increased the yield about tenfold compared to succDMM (see Additional file 1: Table S4). In contrast, *E. coli* BL21 pET28b-ptetO::*sod_gfp*v2 cultivation in TB showed a tremendous boost of sodorifen emission which continuously increased with each day and revealed a 26-fold higher production titer on day 4 when compared to the optimal production condition of the original producer (Fig. 2; see Additional file 1: Table S4 for a complete overview of produced sodorifen amounts).

**Fig. 4:**
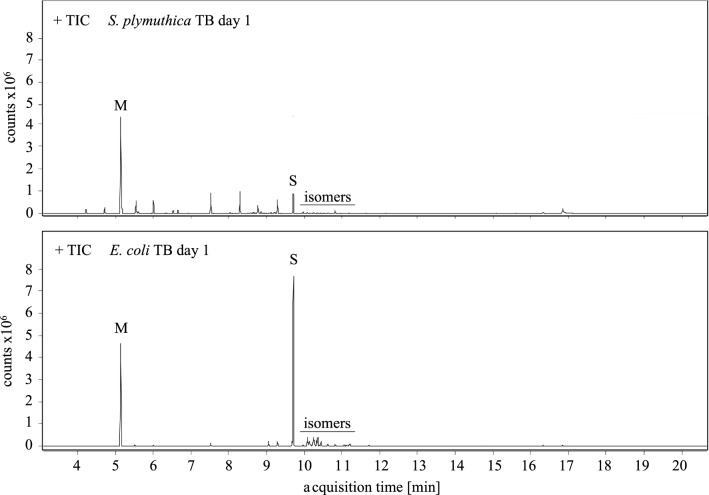
GC–MS total ion chromatograms of *S. plymuthica* WS3236 (top) and *E. coli* pET28b-ptetO::*sod*_*gfp*v2 (bottom) VOCs produced in TB medium within the first 24 h hours of cultivation. Mesitylene (M) was added as an internal standard with a concentration of 250 µg/mL. The amount of sodorifen (S) produced heterologously by *E. coli* is significantly higher combined with better purity compared to *S. plymuthica*

GC and GC–MS analysis revealed that *S. plymuthica* WS3236 produces a variety of different VOCs in comparable amounts (Fig. 4) as described previously [4]. In contrast to this result, *E. coli* pET28b-ptetO::*sod_gfp*v2 exhibits a very clean VOC profile with sodorifen as the major VOC (Fig. 4). Only in fermentations carried out in LB we found a second compound that significantly added to the spectrum of emitted substances and was subsequently identified as indole by NMR analysis, a VOC known to be produced by *E. coli* in high quantities [31, 32]. NMR measurements of the crude extract from the sampled head space of the heterologous *E. coli* fermentation in TB on day 2 showed high purity of sodorifen without any further purification (see Additional file 1: Figure S6). The relative production titers analyzed by GC–MS based on relative peak areas compared to mesitylene as an internal standard with defined concentration were nicely consistent across analyses of replicates (e.g., ranging from 232 to 318 on day one and from 1003 to 1018 at maximum production on day 3 or 4). However, isolated yields of sodorifen derived by head-space sampling varied more significantly (e.g., ranging from 6.4 to 7.9 mg within 24 h at maximum production on days 3 or 4). This is a direct consequence of the volatility of sodorifen, requiring careful removal of the organic solvent under reduced pressure (pentane, used for extraction of the target compound from activated charcoal, see “Methods”). The total amount of combined isolated sodorifen head-space extract from TB fermentation over a time-course of 4 days thus ranged from 13.4 up to 25.5 mg. The developed expression system is therefore suitable for the production of sufficient amounts of sodorifen by simple head-space sampling thus establishing a reliable supply of the compound.

**Fig. 5:**
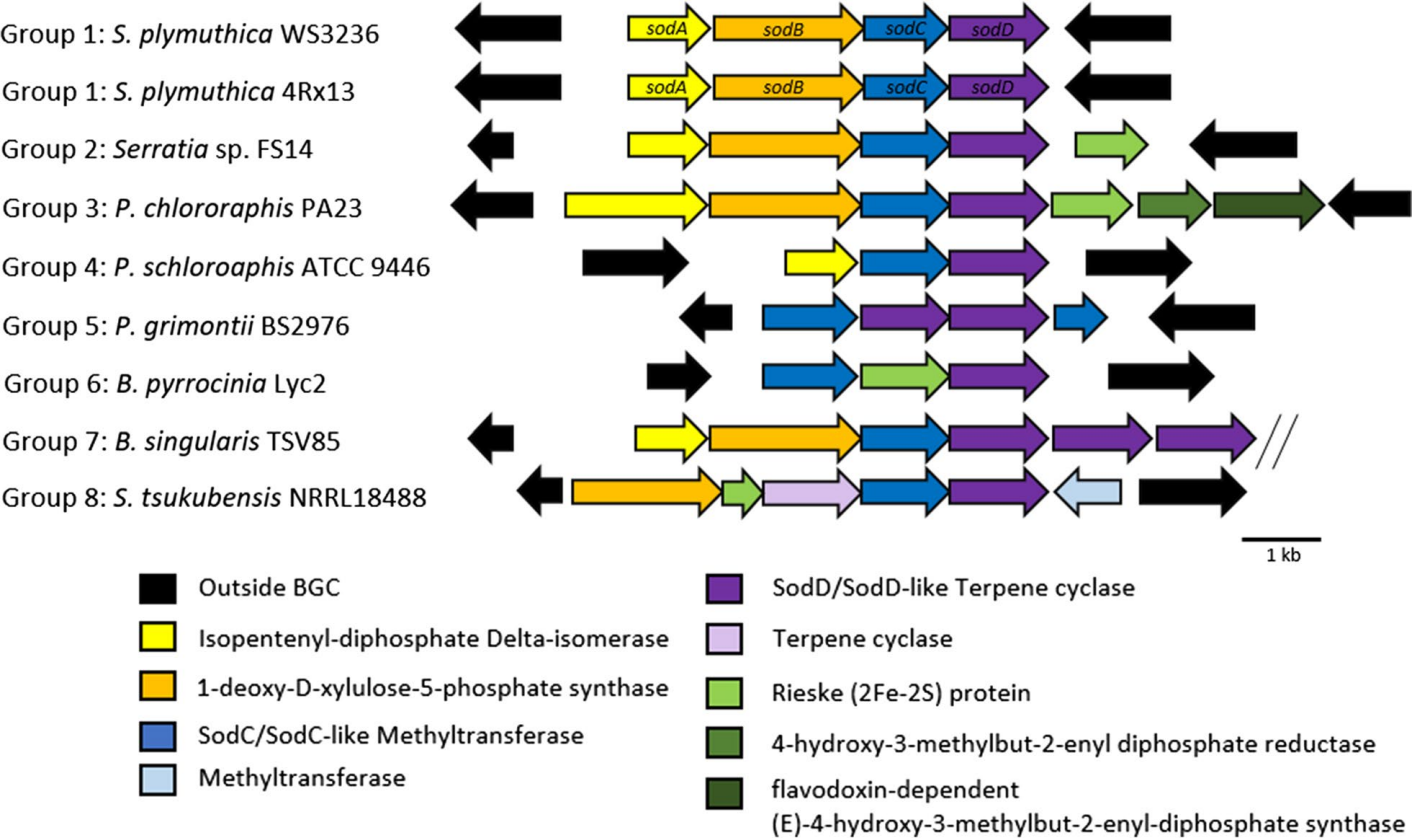
Comparative analysis of *sod* gene clusters throughout the bacterial lineage. A total of 28 *sod*-like gene clusters were identified and categorized into eight groups. Groups are categorized at the inter-species level. All gene clusters except for *B. pyrrocinia* retained a conserved *sodCD* gene synteny. Double lines indicate the edge of the genome sequence contig in *B. singularis* TSV85

Given the conserved nature of the *sod* pathway and the unique rearranged terpene structure that is assembled following the *sod* biosynthetic logic, we aimed at identifying further *sod*-type BGCs by performing a bioinformatic screen of all publicly available microbial genomes within the NCBI and JGI IMG databases using SodC (methyltransferase) and SodD (terpene cyclase) as a query sequence, since these are essential for sodorifen biosynthesis. A total of 28 putative *sod*-like gene clusters were identified in *S. plymuthica*, *Serratia* sp., *Pseudomonas chlororaphis*, *Pseudomonas grimontii*, *Pseudomonas schloroaphis*, *Burkholderia pyrrocinia, Burkholderia singularis* and *Streptomyces tsukubensis* (Additional file 1: Table S2). While a *sod*-like gene cluster has previously been reported in *P. chlororaphis* O6 and *Streptomyces tsukubensis* NRRL18488 [11, 12], we report similar genomic regions in several other species here for the first time. Comparison of the gene synteny allowed the categorization of *sod* clusters into eight groups including differences in encoding one or several oxidoreductase-type enzymes, multiple methyltransferases, or multiple copies of terpene cyclase-type enzymes (Fig. 5). Based on our data, *sod*-like clusters are unique only at the inter-species level. *Serratia* sp. FS14 displayed an intriguing phylogenetic distribution, with the FS14-SodD clustering with the *P. chlororaphis* clade (Additional file 1: Figure S1). Of further interest was the identification of three similar terpene cyclase genes forming an operon within the *B. singularis* TSV85 *sod* cluster. This bioinformatic analysis clearly reveals the presence of further *sod*-type BGCs that are interesting targets for functional expression to obtain further unusual, structurally novel terpenoids.

## Discussion

*Serratia plymuthica* is notorious for producing a wide array of VOCs. Sequencing data of the *S. plymuthica* WS3236 genome was consistent with other *S. plymuthica* strains, in particular the well characterized *S. plymuthica* 4Rx13 (formerly *Serratia odorifera* 4Rx13 [4]), which has a similar genome size and number of CDS. In silico analysis of the encoded BGCs within *S. plymuthica* WS3236 revealed 38 in total, with 9 BGCs belonging to well-characterized natural product families. Comparison of BGCs against *S. plymuthica* 4Rx13 revealed only 6 out of the 38 clusters are shared between the two organisms, highlighting the inter-species BGC variability in the genus *Serratia*.

Most *S. plymuthica* strains investigated so far emit a complex mixture of a large number of different VOCs, most of them produced at very low levels [4], making the functional screening of individual compounds such as sodorifen almost impossible. Schmidt et al. [12] recently developed a successful *E. coli* based system harboring the sodorifen methyltransferase and the terpene cyclase as well as an additional mevalonate pathway-expression plasmid encoding a mevalonate kinase, a phosphomevalonate kinase and a mevalonate pyrophosphate decarboxylase to increase the presence of farnesyl pyrophosphate, the precursor of sodorifen, but were only able to produce low amounts of sodorifen [12]. To further improve the yield of heterologously producible sodorifen, we thus decided to use the entire natural sodorifen production gene cassette under the control of an inducible promoter. We selected the tetracycline-induced promoter P*tet*_*O*_, which has successfully been utilized in the heterologous expression of multiple natural product BGCs [25, 26, 33]. This expression system was also successful in this study and allowed for a constant sodorifen biosynthesis in amounts significantly higher than the natural producer. With this approach, we were able to increase the amount of emitted sodorifen up to 26-fold compared to the optimal production conditions of the original producer *S. plymuthica* WS3236. In addition, we obtained sodorifen in high purity by head-space sampling, thus providing an efficient system for sodorifen production, which sets the stage for further investigations into the biological function of this interesting compound.

Within this work, we furthermore investigated the occurrence of additional candidate sodorifen-type BGCs in bacteria. In silico screening of published genomes led to the identification of 28 *sod* and *sod*-like gene clusters throughout bacteria, which is a significant increase on previously reported numbers [11, 12]. Intriguingly, while *Serratia* sp. FS14 was isolated from plants, similar to other sodorifen producing bacteria, its genome is more closely related to non-plant associated *Serratia marcescens* [34], but still encodes a *sod* gene cluster. The *sod* cluster of *Serratia* sp. FS14 differed significantly compared to other *Serratia*, with a much lower sequence identity (~ 63% FS14 vs WS3236 compared to ~ 96% 4Rx13 vs WS3236) and the presence of a Rieske (2Fe-2S) protein that is similar to those in *P. chlororaphis* (58%) and *B. pyrrocinia* (56%). The phylogenetic clustering of FS14-SodD with homologous proteins from *P. chlororaphis* (Additional file 1: Figure S1) may be the first hint that organisms outside of *S. plymuthica* species are capable of producing sodorifen or sodorifen analogs.

Based on gene synteny analysis, we were able to categorize the *sod*-like clusters into eight groups, which mostly separated at the species level. Differences between these *sod*-like clusters include the presence of one or several oxidoreductase-type enzymes, multiple methyltransferases, or multiple copies of terpene cyclase-type enzymes. This suggests that there are significant modifications to the sodorifen backbone in order to produce a range of analogues, which could be a significant source of novel unusual bacterial terpenes.

## Conclusions

Genome sequencing and bioinformatic analysis revealed that there is a significant BGC diversity within different *S. plymuthica* species. Comparison and phylogenetic analysis of SodD of *Serratia* sp. FS14 indicates this pathway shares some homology with *P. chlororaphis*, raising interesting evolutionary questions about sodorifen biosynthesis. Our in silico analysis furthermore revealed the presence of a diversity of *sod*-type BGCs across phylogenetically diverse bacteria. Significant genetic variability (e.g., additional methyltransferases, terpene cyclases and oxidoreductase enzymes) between these pathways indicates that there likely is a plethora of unique and structurally intriguing sodorifen analogs awaiting discovery. Using DiPaC, we quickly integrated the native four gene containing sodorifen cluster *sodABCD* into a vector under the control of one single promoter upstream of *sodA*, thereby mimicking the transcription setting in the original producer. This ensures that the relevant enzymes of the MEP-pathway SodA and SodB are overproduced synchronously with SodC and SodD, which finally transform SAM and FPP into sodorifen. By screening a variety of different culture conditions, sodorifen production was increased up to 26-fold compared to the original producer, accompanied by significantly improved purity in the crude extract. The results of this work pave the way for future studies investigating the biological role and function of sodorifen.

## Methods

### Bacterial culturing, strains and plasmids

Bacterial strains and plasmids generated in this study are listed in Table 1. *S. plymuthica* WS3236 was obtained from the Weihenstephaner collection of microorganisms (Chair of Microbial Ecology, Technical University of Munich Freising, Germany). *S. plymuthica* WS3236 was cultivated in nutrient broth (Carl Roth, Germany) at 28 °C (liquid cultures) or 30 °C (agar plates). Liquid cultures were incubated while shaking at 200 rpm. Under routine culture conditions unless otherwise specified, *E. coli* strains were grown in lysogeny broth (LB) medium (Carl Roth, Germany) supplemented with 50 µg/mL kanamycin.

**Table 1:** Bacterial strains and plasmids used in this study

	Description	Reference or source
Strains		
*Escherichia coli* DH5α	Host strain for cloning	NEB
*Escherichia coli* BL21	Heterologous expression strain	NEB
*Serratia plymuthica* WS3236	Native producer of sodorifen	ZIEL Institute Culture Collection
Plasmids		
pET28b-ptetO-*gfp*v2 (6029 bp)	Tetracycline inducible expression plasmid, ColE1, Kan^R^, *gfp* reporter gene downstream of promotor	This study
pET28b-ptetO::*sod*_*gfp*v2 (11,124 bp)	pET28b-ptetO_*gfp*v2 with *sodABCD* cloned as single fragment between P*tet*_*O*_ and *gfp*	This study

### Genomic DNA extraction, whole genome sequencing (WGS) and assembly

Extraction of high-molecular weight genomic DNA was performed as described by Micallef [35] and Greunke et al. [25] with few alterations. After culturing, cells were placed at 4 °C for 24 h to stop cell division and DNA replication prior to DNA extraction. After centrifugation (10 min at 10,000×*g*), the cell pellet was resuspended in 5 mL lysis buffer (25 mM EDTA, 0.3 M sucrose, 25 mM Tris–HCl [pH 7.5]). The cells were freeze-thawed three times (liquid nitrogen/50 °C) to promote cell lysis. Lysozyme (Sigma-Aldrich) was added to a final concentration of 1 mg/mL and RNase (Carl Roth, Germany) to a final concentration of 10 μg/mL followed by an incubation at 37 °C for 60 min. Proteinase K (Amresco, USA) was added to a final concentration of 0.5 mg/mL followed by the addition of SDS to a final concentration of 1% (w/v). The cell mixture was incubated at 37 °C for 30 min followed by 55 °C for 30 min. NaCl was added to a final concentration of 1.3 M, and cetyltrimethylammonium bromide (CTAB) [10% (w/v) CTAB (Sigma-Aldrich) in 0.7 M NaCl] to a final concentration of 1% (v/v) CTAB solution, with the mixture incubated at 65 °C for 10 min. A total of 1 vol of chloroform:isoamyl alcohol (24:1) was added to the cell lysis solution, mixed by inversion and incubated on ice while shaking for 30 min. The aqueous phase was removed and phenol–chloroform–isoamylalcohol (25:24:1) extraction was performed twice before the addition of 0.6 vol of isopropanol. The DNA cell pellet was washed with ice cold 70% ethanol and finally dissolved in 0.1× TE buffer (1 mM Tris–HCl, 0.1 mM EDTA [pH 8.0]). The quality of gDNA was analyzed by gel electrophoresis using 0.7% (w/v) agarose gels and quantified with a P330 NanoPhotometer (Implen, Germany).

The whole genome of *S. plymuthica* WS3236 was generated at the DOE Joint Genome Institute (JGI) using the Pacific Biosciences (PacBio) sequencing technology [36]. A > 10 kb PacBio SMRTbell™ library was constructed and sequenced on the PacBio RS2 platform, which generated 78,182 filtered subreads totaling 307,584,001 bp. The raw reads were assembled using HGAP (smrtanalysis/2.3.0 p5, HGAP 3) [37]. The final draft assembly contained 1 contig in 1 scaffold, totaling 5,349,225 bp in size. The input read coverage was 45.2×. The genome sequencing QC report can be found as Additional file 2.

### Bioinformatic and phylogenetic analysis

In silico analysis of the BGCs present in the *S. plymuthica* WS3236 genome was performed using AntiSMASH (Version 4) [27]. The presence of the *sod* cluster throughout all available genome sequences against the NCBI and JGI IMG databases (as of October 2018) was searched using the *S. plymuthica* SodC (methyltransferase; locus_tag: Ga0236286_4400) and SodD (terpene cyclase; locus_tag: Ga0236286_4399) as query sequences. BlastP analysis with these query sequences was performed against the NCBI non-redundant and JGI Integrated Microbial Genomes System [38] databases. All hits consisting of adjacent methyltransferase and terpene cyclase genes were categorized as possible *sod*-type clusters. Phylogenetic analysis was performed using the Phylogeny.fr online tool with ‘one click’ analysis [39, 40]. All sequences were stored and visualized using the Geneious Software Package.

### Direct pathway cloning of the *sod* cluster

The 4.6 kb *sod* cluster of *S. plymuthica* WS3236 was cloned in one piece into pET28b-ptetO using SLIC-mediated DiPaC [26]. Linearized PCR amplicons of both the vector backbone and the *sodABCD* fragment were generated using PCR with Q5 polymerase (NEB) under standard conditions with 50 ng gDNA per 25 µL reaction setup (see Additional file 1: Table S3 for primer sequences and chapter 2.1 for PCR setup). At the 5′ end of the cluster specific primer pair, 22 bp homology sequences consistent with the terminal region of the PCR generated vector were added. After purification, the fragments were assembled with T4-DNA polymerase and 8 µL of the reaction mixture was chemically transformed into *E. coli* DH5α. Positive pET28b-ptetO::*sod*_*gfp*v2 clones were selected by Taq screening PCR with primers binding *sodD* and the T7 terminator (see Additional file 1: Table S3 for primer sequences and chapter 2.2 for PCR setup) and confirmed using restriction digest and Sanger sequencing of the integration sites.

### Bacterial fermentation for sodorifen production

All fermentations were performed in 3 L scale using a BIOSTAT A plus fermenter (type 8843812, Sartorius Stedim Biotech) and the volatile compounds were collected at the gas outlet with a filter consisting of 0.5 g pure activated charcoal between two VitraPOR glass filters (porosity 00, ROBU, Germany) in a plastic centrifuge column (Pierce 5 mL, Thermo Scientific, Germany). 3 L cultures were inoculated 1:50 from overnight pre-cultures grown in LB medium and 300 µL of Antifoam SE-15 (Sigma-Aldrich/Merck, Germany) was added. *S. plymuthica* WS3236 cultures were grown in terrific broth (TB) medium (Carl Roth, Germany) and modified Davis and Mingioli succinate minimal medium (succDMM: 7 g/L K_2_HPO_4_*3 H_2_O, 3 g/L KH_2_PO_4_, 1 g/L (NH_4_)_2_SO_4_, 0.5 g/L sodium citrate dihydrate, 6.49 g/L succinate, 0.1 g/L MgSO_4_*7 H_2_O, pH 6.2 with NaOH) at 30 °C with 200 rpm stirring. Trapping of volatile compounds started directly after inoculation and the filters were exchanged every 24 h for up to 4 days. 300 µL of Antifoam SE-15 was again added after 40 h and 72 h.

For the heterologous expression of sodorifen, *E. coli* BL21 cells were chemically transformed with pET28b-ptetO::*sod_gfp*v2. Expression was carried out in LB, TB and succDMM, each supplemented with 50 mg/L kanamycin. After inoculation, cells were grown at 37 °C at 200 rpm for 5 h before the temperature was lowered to 16 °C and expression induced with 708 µg/L tetracycline. Volatile compound collection was started immediately after induction and the filters were exchanged every 24 h for up to 4 days. 300 µL of Antifoam SE-15 was again added after 48 h.

After fermentation, the active charcoal was extracted twice with 2 mL pentane, which was filtered through a 0.45 µm PTFE syringe filter (Fisher Scientific, Germany) to remove remaining coal pieces before evaporation in vacuo. The residues were weighed and re-suspended in pentane or CDCl_3_ for further measurements. Mesitylene was used as an internal standard for GC analysis with a concentration of 250 µg/mL.

### GC–(MS) and NMR analysis of bacterial head-space extracts

Analytical gas chromatography was performed at a HP 6890 Series GC (Agilent, stationary phase: HP-5 column, poly-dimethyl/diphenyl-siloxane, 95/5) with a flame ionization detector using the following temperature profile: 60 °C (hold 3 min), then 15 °C/min to 250 °C (hold 5 min). The amounts of mesitylene and sodorifen were determined by peak area integration. Mass spectrometric analysis was performed with electron impact ionization (EI, 70 eV) on a Agilent HP 6890 Series GC–MS (Agilent, stationary phase: HP-5MS column, poly-dimethylsiloxane, 30 m, mass detection: Agilent 5973 Network Mass Selective Detector) using the same temperature profile as for analytical GC. NMR spectra of sodorifen and the *E. coli*-derived indole were directly recorded from head-space samples on a Bruker AVHD500 and a Bruker AV500-cryo spectrometer in CDCl_3_ (see Additional file 1: Figure S6).

## Additional files

**Additional file 1.** Antismash and Cluster Finder results; organisation of *sod*-like BGCs into cluster types; phyogenetic analysis of SodC and SodD; methods for the cloning and expression of the *sod* BGC; results of the heterologous expression of the *sod* cluster; GC–MS spectrum of sodorifen; NMR spectra of raw head-space samples of *E. coli* harbouring pET28b-ptetO::*sod_gfp*v2 expression vector.

**Additional file 2.** JGI whole-genome sequencing report of *S. plymuthica* WS3236.

## Abbreviations

BGC: biosynthetic gene cluster
CTAB: cetyl trimethylammonium bromide
DiPaC: direct pathyway cloning
DXP: 1-deoxy-D-xylulose-5-phosphate
EDTA: ethylenediaminetetraacetic acid
*E. coli*: *Escherichia coli*
FPP: farnesyl pyrophosphate
GC: gas chromatography
GC–MS: gas chromatography-mass spectrometry
GFP: green fluorescent protein
IPP: isopentenyl-diphosphate
LB: lysogeny broth
MEP: non-mevalonate pathway
NMR: nuclear magnetic resonance
PCR: polymerase chain reaction
PTFE: polytetrafluoroethylene
SAM: *S*-adenosyl methionine
*S. plymuthica*: *Serratia plymuthica*
succDMM: modified Davis and Mingioli minimal medium containing 55 mM succinate
TB: terrific broth
TE: Tris-EDTA
Tris: tris(hydroxymethyl)aminomethane
VOC: volatile organic compound
WGS: whole genome sequencing
