# Supplementary material for: Direct pathway cloning of the sodorifen biosynthetic gene cluster and recombinant generation of its product in E. coli
Source: Microb Cell Fact. 2019 Feb 7;18:32. doi: 10.1186/s12934-019-1080-6 (PMC6366047; doi:10.1186/s12934-019-1080-6)
Supplement: Supplementary file 1 — Additional file 1. Antismash and Cluster Finder results; organisation of sod-like BGCs into cluster types; phyogenetic analysis of SodC and SodD; methods for the cloning and expression of the sod BGC; results of the heterologous expression of the sod cluster; GC–MS spectrum of sodorifen; NMR spectra of raw head-space samples of E. coli harbouring pET28b-ptetO::sod_gfpv2 expression vector. [file 12934_2019_1080_MOESM1_ESM.pdf]

Direct Pathway Cloning of the Sodorifen Biosynthetic Gene Cluster and Recombinant Generation of its Product in *E. coli*

Elke R. Duell[1†], Paul M. D'Agostino[1†], Nicole Shapiro[2], Tanja Woyke[2], Thilo M. Fuchs[3,4] and Tobias A. M. Gulder[1,5]*

[1]Biosystems Chemistry, Department of Chemistry and Center for Integrated Protein Science Munich (CIPSM), Technical University of Munich, Lichtenbergstraße 4, 85748 Garching, Germany.

[2]Department of Energy, Joint Genome Institute, 2800 Mitchell Drive, Walnut Creek, CA 94598, USA

[3]ZIEL Institute for Food & Health, Lehrstuhl für Mikrobielle Ökologie, Department biowissenschaftliche Grundlagen, Technical University of Munich, Munich, Germany

[4]Friedrich-Loeffler-Institut, Institut für Molekulare Pathogenese, Jena, Germany

[5]Technische Universität Dresden, Chair of Technical Biochemistry, Bergstraße 66, 01602 Dresden, Germany.

[†] Contributed equally

[*]Correspondence: tobias.gulder@ch.tum.de

SUPPORTING INFORMATION

Table of contents

1 Antismash and Cluster Finder results..2

2 Direct Pathway Cloning of the *sod* cluster...6

2.1 Q5 Polymerase PCR setup..6

2.2 Taq Polymerase PCR setup...6

3 Expression of the *sod* cluster..7

1 Antismash and Cluster Finder results

Table S1: Antismash results of the *S. plymuthica* WS3236 genome.

Cluster 1	Nrps	152180	221285	Ravidomycin_biosynthetic_gene_cluster (5% of genes show similarity)	BGC0000263_c1
Cluster 2	Cf_putative	277500	283014	-	-
Cluster 3	Cf_putative	343838	351083	-	-
Cluster 4	Cf_putative	452077	458216	-	-
Cluster 5	Cf_fatty_acid	530237	551451	-	-
Cluster 6	Nrps	585530	638119	Turnerbactin_biosynthetic_gene_cluster (30% of genes show similarity)	BGC0000451_c1
Cluster 7	Cf_fatty_acid	1101356	1122603	-	-
Cluster 8	Cf_putative	1225164	1234949	PM100117_/_PM100118_biosynthetic_gene_cluster (21% of genes show similarity)	BGC0001359_c1
Cluster 9	Otherks-T1pks-Pufa-Nrps	1309016	1391220	Zeamine_biosynthetic_gene_cluster (95% of genes show similarity)	BGC0001056_c1
Cluster 10	Cf_putative	1517998	1528737	-	-
Cluster 11	Nrps	1638611	1682552	Lipopolysaccharide_biosynthetic_gene_cluster (16% of genes show similarity)	BGC0000772_c1
Cluster 12	Cf_saccharide	2079656	2111872	Lipopolysaccharide_biosynthetic_gene_cluster (27% of genes show similarity)	BGC0000776_c1
Cluster 13	Cf_putative	2307169	2314735	-	-
Cluster 14	Cf_putative	2360781	2372066	O&K-antigen_biosynthetic_gene_cluster (3% of genes show similarity)	BGC0000780_c1
Cluster 15	Cf_putative	2400546	2408494	-	-
Cluster 16	Cf_putative	2768674	2773791	-	-
Cluster 17	Arylpolyene-Siderophore	3060557	3126399	APE_Ec_biosynthetic_gene_cluster (73% of genes show similarity)	BGC0000836_c1
Cluster 18	Nrps	3174907	3226855	Vulnibactin_biosynthetic_gene_cluster (12% of genes show similarity)	BGC0000460_c1
Cluster 19	Cf_putative	3389902	3400844	-	-
Cluster 20	T1pks-Nrps	3402732	3450570	Rishirilide_B_biosynthetic_gene_cluster (7% of genes show similarity)	BGC0001179_c1
Cluster 21	Cf_putative	3501638	3513935	-	-
Cluster 22	Cf_putative	3592222	3597199	-	-
Cluster 23	Cf_saccharide	3890501	3917908	Stewartan_biosynthetic_gene_cluster (57% of genes show similarity)	BGC0000763_c1

Cluster 24	Cf_saccharide	3920935	3942988	O-antigen_biosynthetic_gene_cluster (30% of genes show similarity)	BGC0000787_c1
Cluster 25	Cf_putative	3988487	3998306	-	-
Cluster 26	Thiopeptide	4020188	4046677	O-antigen_biosynthetic_gene_cluster (14% of genes show similarity)	BGC0000781_c1
Cluster 27	Cf_fatty_acid	4085148	4106422	Taxlllaid_biosynthetic_gene_cluster (33% of genes show similarity)	BGC0001133_c1
Cluster 28	Cf_putative	4113644	4134661	Prodigiosin_biosynthetic_gene_cluste r (82% of genes show similarity)	BGC0000258_c1
Cluster 29	Cf_putative	4144920	4160325	-	-
Cluster 30	Cf_fatty_acid	4219983	4240936	-	-
Cluster 31	Cf_putative	4388170	4393085	-	-
Cluster 32	Cf_putative	4415768	4422464	-	-
Cluster 33	Terpene	4586723	4607877	Sodorifen_biosynthetic_gene_cluster (100% of genes show similarity)	BGC0001361_c1
Cluster 34	Cf_putative	4660496	4673055	-	-
Cluster 35	Cf_putative	4742629	4766296	-	-
Cluster 36	Cf_putative	4840226	4854533	-	-
Cluster 37	Cf_putative	5133490	5142678	Polysaccharide_B_biosynthetic_gene_cluster (6% of genes show similarity)	BGC0001411_c1
Cluster 38	Cf_fatty_acid-Cf_saccharide	5297309	5338267	Tallysomycin_biosynthetic_gene_clus ter (5% of genes show similarity)	BGC0001048_c1

**Table S2: Organisms and accession number of organisms harboring *sod*-like BGCs.**		
**Organism**	**Genome Accession**	**Gene Cluster Group**
Serratia plymuthica 4Rx13	CP006250	1
Serratia plymuthica A30	AMSV01000037	1
Serratia plymuthica AS9	CP002773	1
Serratia plymuthica NBRC 102599	NZ_BCTU01000010	1
Serratia plymuthica PRI-2C	NZ_CP015613	1
Serratia plymuthica RVH1	NZ_ARWD01000001	1
Serratia plymuthica S13	CP006566	1
Serratia plymuthica 3Re4-18	NZ_CP012097	1
Serratia plymuthica 3Rp8	NZ_CP012096	1
Serratia plymuthica 4Rx5	NZ_PESE01000001	1
Serratia plymuthica HRO-C48	NZ_LTDN01000056	1
Serratia plymuthica strain V4	CP007439	1
Serratia plymuthica WS3236	2773857786 (JGI)	1
Serratia sp. AS12	CP002774	1
Serratia sp. AS13	CP002775	1
Serratia sp. FS14	CP005927	2
Pseudomonas chlororaphis O6	NZ_CM001490	3
Pseudomonas chlororaphis ATCC 13985	NZ_LT629738	3
Pseudomonas chlororaphis PA23	NZ_CP008696	3
Pseudomonas chlororaphis subsp. *aureofaciens* NBRC 3521	NZ_BBQB01000002	3
Pseudomonas chlororaphis subsp. *aureofaciens* CD	NZ_LHVB01000012	3
Pseudomonas chlororaphis subsp. *aureofaciens* LMG 1245	LHVA01000023	3
Pseudomonas chlororaphis subsp. *chlororaphis* GP72	NZ_AHAY01000123	3
Pseudomonas schloroaphis ATCC 9446	NBAT01000014	4
Pseudomonas grimontii BS2976	FNKM01000002	5
Burkholderia pyrrocinia Lyc2	JPWP01000010	6
Burkholderia singularis TSV85	LOWA01000011	7
Streptomyces tsukubensis NRRL18488	AJSZ01000838	8

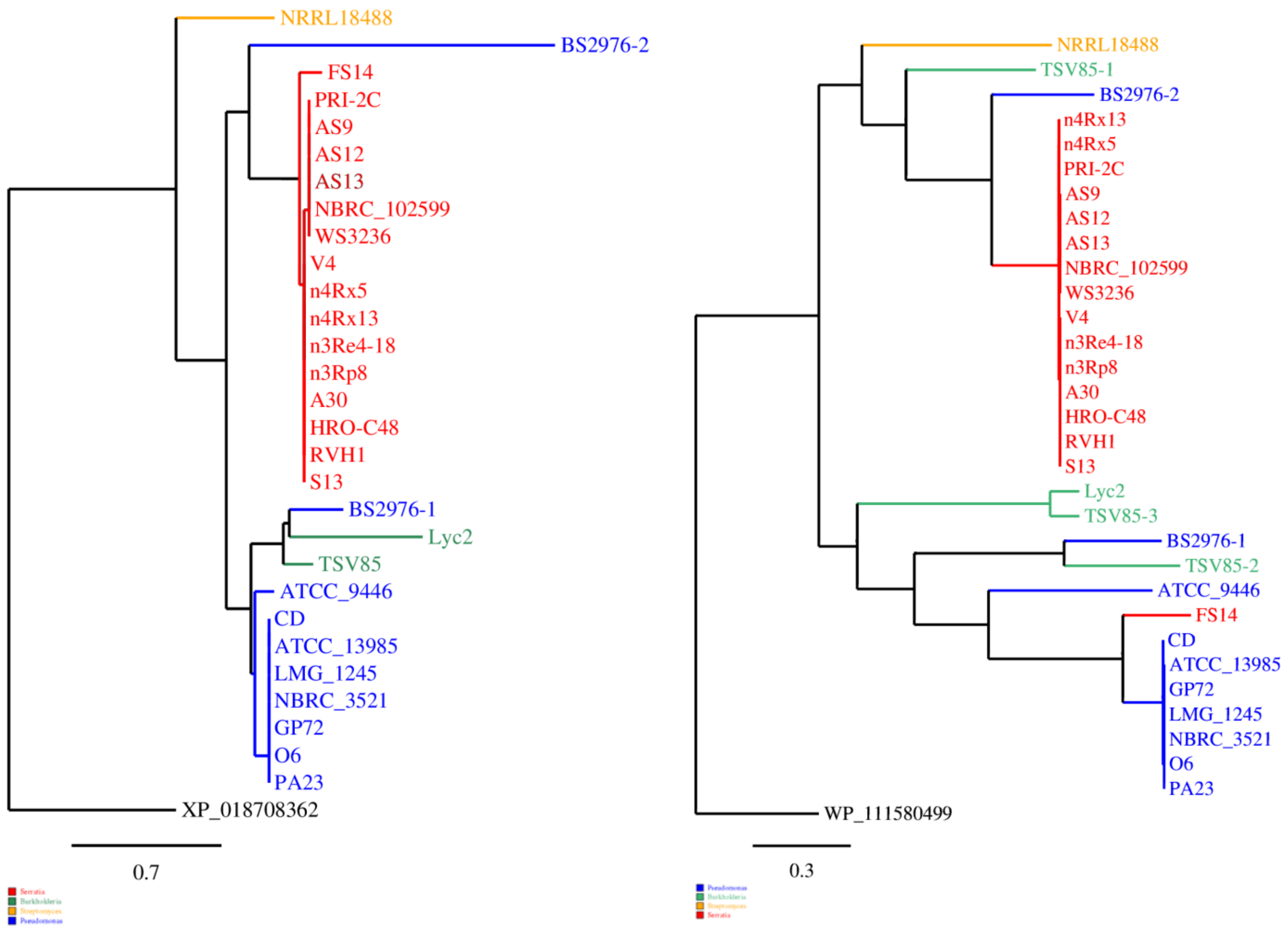

Figure S1: Phylogenetic analysis of SodC (A) and SodD (B). Organism titles can be linked to species list in Table S2. Outgroups are in black and included XP_018708362 (SodC tree) and WP_111580499 (SodD tree). Branches and strain names are colored according to genus: *Serratia*, red; *Pseudomonas*, blue; *Burkholderia*, green; *Streptomyces*, orange.

2 Direct Pathway Cloning of the *sod* cluster

**Table S3: List of primers.**		
Name	Sequence (5' → 3')	Description
C-GFP_for_1	CATGGTTAGCAAAGGTGAAG	amplification of the pET28b-ptetO_*gfp*v2 backbone
spec-ptet-R	GGTCGATCCTCTTCTCTATC	
SP-sod::ptet_for_1	gtgatagagaagaggatcgaccATGCTGATCCTCGTTGATGACAAAG	amplification of *sod* cluster with 22 bp homology sequences
SP-sod::C-GFP_rev_1	TTCTTCACCTTTGCTAACCATGttagcgccgcccgtactg	
sodD_screen_for_1	GAAGTATTAAATGGACCGC	colony screening of pET28b-ptetO::*sod_gfp*v2
pRSET-RPnew	GGGTTATGCTAGTTATTGC	
screen_ptet_F	TGCGCTGTTAATCACTTTAC	sequencing pET28b-ptetO::*sod_gfp*v2
screen_GFP_R	TTACCGTTGGTCGCATCACC	

2.1 Q5 Polymerase PCR setup

A standard 25 µL PCR reaction batch for long-amplicon cycling reactions consisted of: 1x Q5 reaction buffer, 200 µM deoxynucleotide triphosphates, 500 nM of forward and reverse primer, 50 ng gDNA template and 0.01 U/µL Q5 High-Fidelity DNA polymerase (NEB) were mixed. Cycling was conducted using a T100 Thermal Cycler (Biorad) as follows: 1.) Initial denaturation, 98 °C for 1 min; 2.) Denaturation, 98 °C for 10 sec; 3.) Annealing, 72 °C for 20 sec; 4.) Extension, 72 °C for 2 min 30 sec; steps 2.) to 4.) were repeated in total for 30 cycles; 5.) Final extension, 72 °C for 5 min, and 6.) End phase, 16 °C.

2.2 Taq Polymerase PCR setup

Colony screening PCRs were performed using *Taq* DNA polymerase (NEB). Clones were picked and resuspended in 50 µL of pure water and examined in a 25 µL PCR batch composed as follows: *Taq* buffer (10 mM Tris-HCl, 1.5 mM $MgCl_2$, 50 mM KCl, pH 8.3 at 25 °C), 4% DMSO, 100 µM deoxynucleotide triphosphates, 200 nM of forward and reverse primer, 5 µL DNA template (bacterial suspension in water) and *Taq* DNA polymerase (0.025 U/µL, NEB) were mixed. Optimal cycling parameters were set as follows: 1.) Initial denaturation, 95 °C for 5 min; 2.) Denaturation, 95 °C for 45 sec; 3.) Annealing, 48 °C for 30 sec; 4.) Extension, 72 °C for 65 sec; steps 2.) to 4.) were repeated in total 34 times; 5.) Final extension, 72 °C for 5 min, and 6.) End phase, 16 °C.

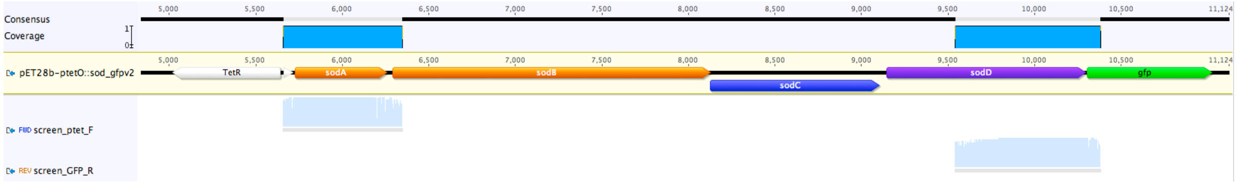

Figure S2: Sequencing of *sodA-D* integration into pET28b-ptetO::*gfp*v2.

3 Expression of the *sod* cluster

Table S4: Quantitative amounts of sodorifen and pre-sodorifen produced during fermentation.

strain	medium	day	peak area pA*s	
			mesitylene[1]	sodorifene
S. plymuthica WS3236	succDMM	1	257.9	12.6
		2	264.0	9.1
		3	267.4	2.7
		4	266.7	1.6
	TB	1	272.9	38.7
		2	272.4	24.4
		3	261.7	4.0
		4	263.4	1.7
E. coli BL21 pET28b-ptetO::*sod_gfp*v2	succDMM	1	267.0	10.8
		2	261.6	36.5
		3	267.8	26.1
		4	262.8	15.9
	TB	1	254.4	317.7
		2	262.6	765.2
		3	265.9	887.9
		4	267.9	1003.0
	LB	1	263.9	180.0
		2	268.9	252.2
		3	268.7	215.3
		4	264.6	105.5

[1]Mesitylene was added as internal standard with a concentration of 250 µg/mL.

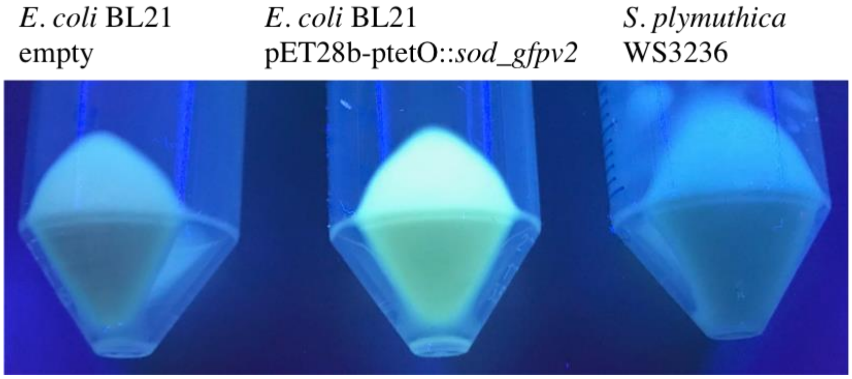

Figure S3: Cell pellets harvested after 24 h cultivation in TB medium. Only the induced heterologous expression strain *E. coli* BL21 pET28b-ptetO::*sod_gfpv2* shows fluorescence caused by the expression of the GFP marker placed downstream of the *sod* genes.

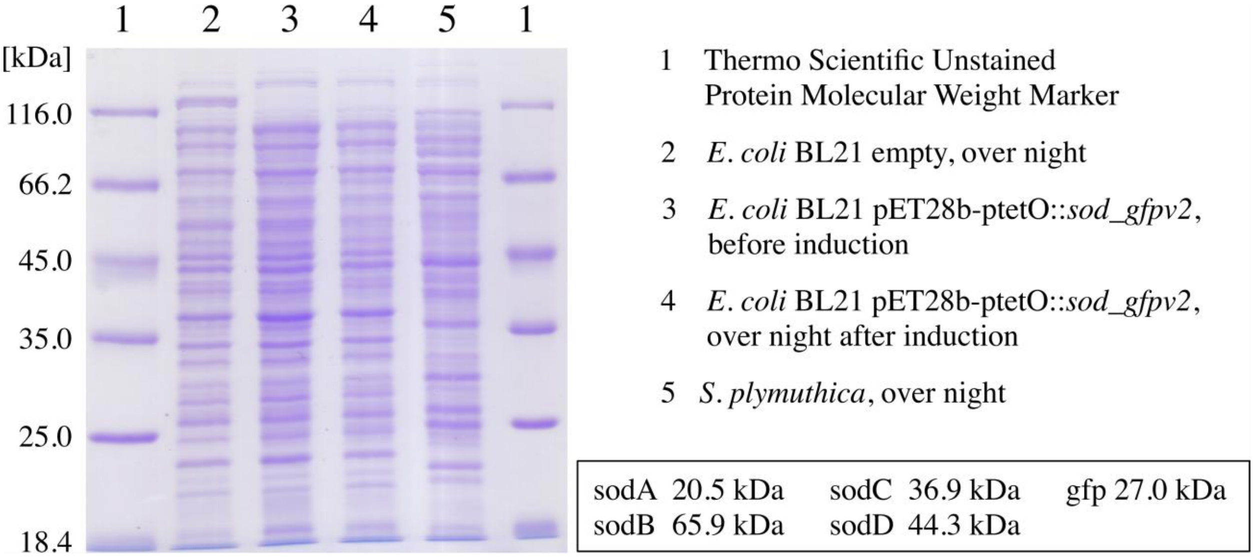

Figure S4: SDS-PAGE analysis of *E. coli* BL21 pET28b-ptetO::*sod_gfpv2* cells before (lane 3) and after induction (lane 4) in comparison to empty *E. coli* BL21 (lane 2) and *S. plymuthica* (lane 5) cells. Due to the usage of the P*tet*$_O$ promoter, no significant protein overexpression can be detected.

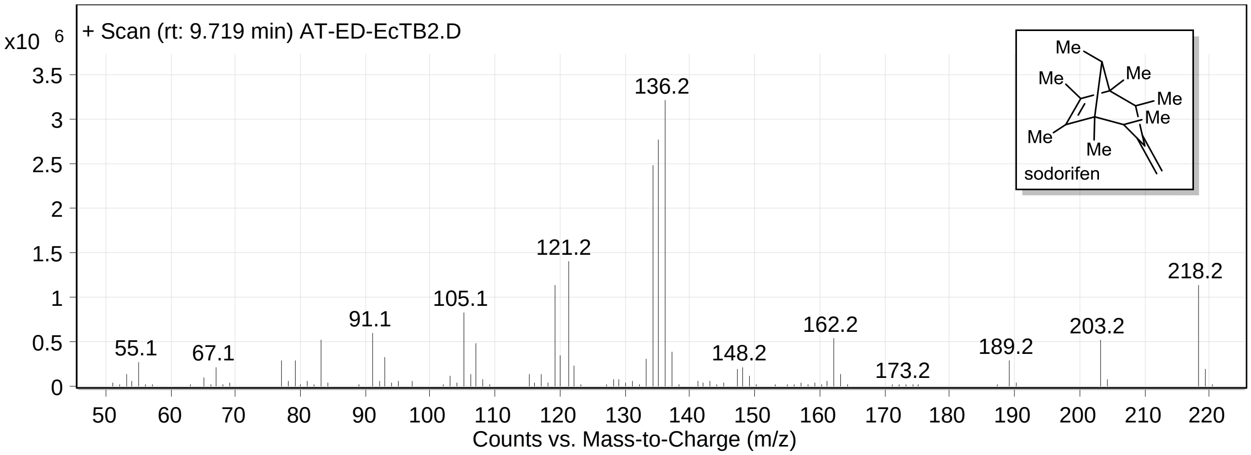

Figure S5: GC-MS spectrum of sodorifen (m/z = 218.20).

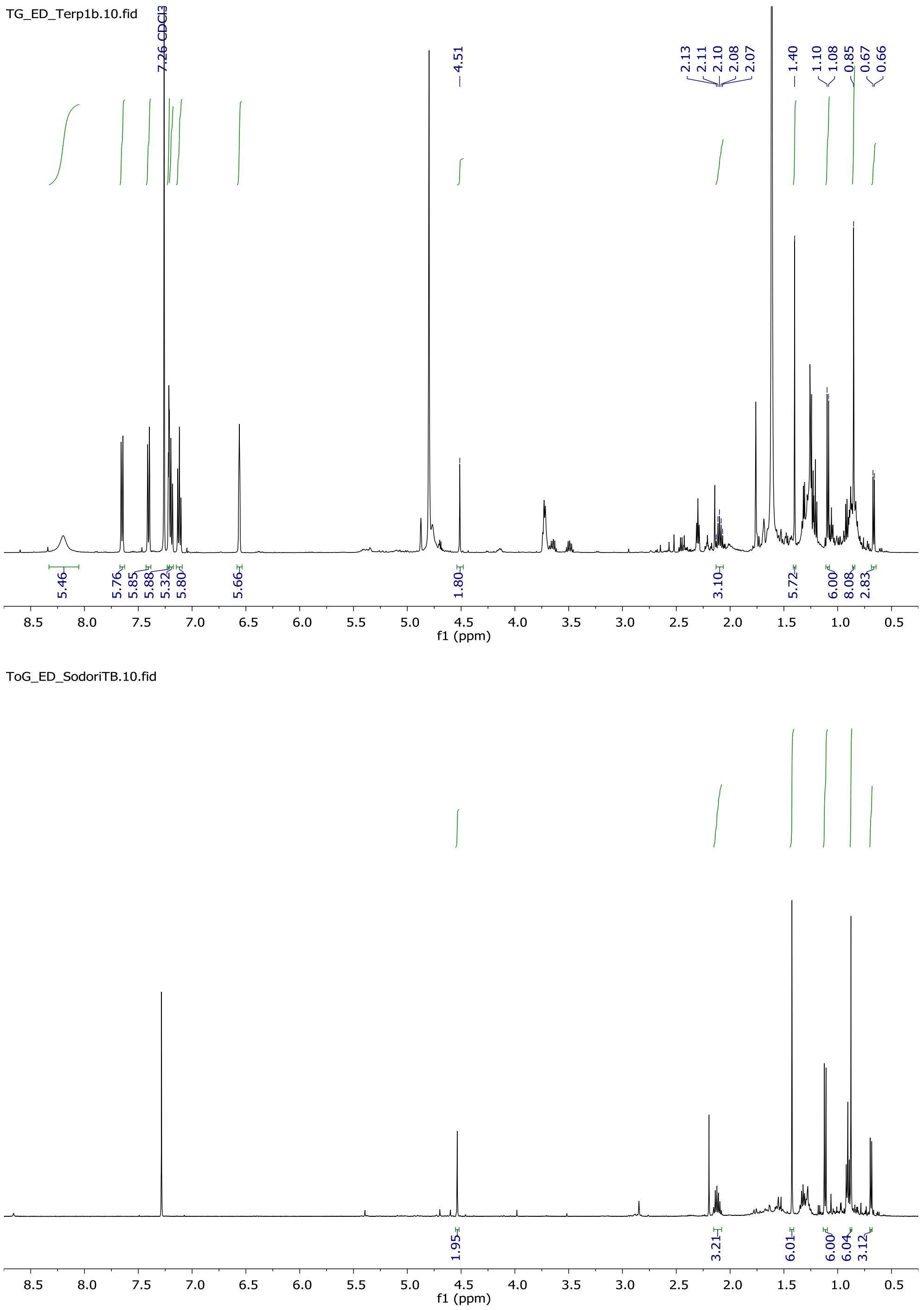

Figure S6: NMR spectra of raw head-space samples of *E. coli* pET28b-ptetO::*sod_gfp*v2 grown in LB (top) and TB (bottom) medium. In the LB-derived top spectrum, the ^{1}H signals of the *E. coli* VOC indole (7 H signals from 8.25 to 6.50 ppm) indicate an about 10-fold excess of indole in the sample when compared to sodorifen (all other integrated ^{1}H signals; relative amounts calculated based on, e.g., the two protons of the exo-methylene unit at 4.5 ppm). The TB-derived bottom spectrum shows almost exclusive formation of sodorifen in high purity.
