# Supplementary material for: Direct pathway cloning of the sodorifen biosynthetic gene cluster and recombinant generation of its product in E. coli
Source: Microb Cell Fact. 2019 Feb 7;18:32. doi: 10.1186/s12934-019-1080-6 (PMC6366047; doi:10.1186/s12934-019-1080-6)
Supplement: Supplementary file 2 — Additional file 2. JGI whole-genome sequencing report of S. plymuthica WS3236. [file 12934_2019_1080_MOESM2_ESM.pdf]

1. Project Information

Program	Microbial/CSP 2017
Sequencing Project ID	1176531
Sequencing Project Name	Serratia plymuthica WS3236

2. Read Statistics

Metric	Raw Reads	Filtered Subreads	Error Corrected Reads
Reads	421,168	78,182	11,725
Bases	1,001,968,894	307,584,001	72,490,082
Average Read Length	$2,379.0 \pm 3,528.4$	$3,934.2 \pm 3,514.5$	$6,182.5 \pm 4,218.7$
Reads >5kbp	60,760	19,569	6,913
Bases, reads >5 kbp	591,145,838	180,537,522	64,177,899
Avg Read Length, reads >5kbp	$9,729.2 \pm 3,814.6$	$9,225.7 \pm 2,985.7$	$9,283.7 \pm 2,378.6$

3. Assembly Statistics

The filtered subreads were assembled using PacBio's HGAP assembler.
Assembly version: smrtanalysis/2.3.0_p5, HGAP 3

Scaffold total	1
Contig total	1
Scaffold sequence length (bp)	5,349,225
Contig sequence length (bp)	5,349,225
Scaffold N/L50	1/5349225
Contig N/L50	1/5349225
Largest Contig (bp)	5,349,225
Number of scaffolds >50 kb	1
Percent of genome in scaffolds >50 kb	100.00%
Percent of reads assembled	83.32%

4. Assembly QC Results

Prodigal was used to predict cds on each scaffold and the output protein sequences were aligned to NCBI nr using LAST. Taxonomic information was extracted from the alignments and used to color-code scaffold GC content histograms.

Kingdom

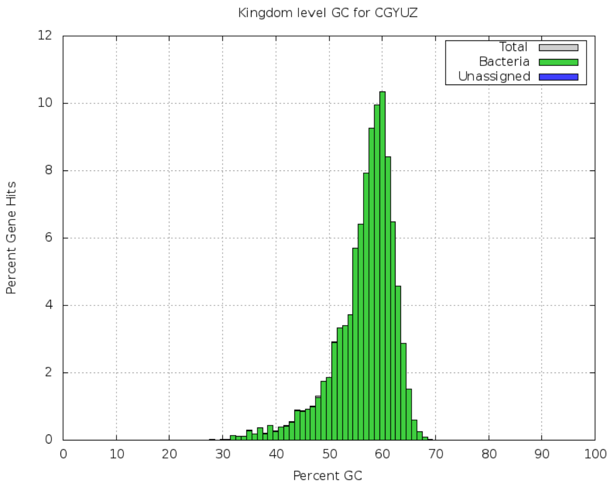

Phylum

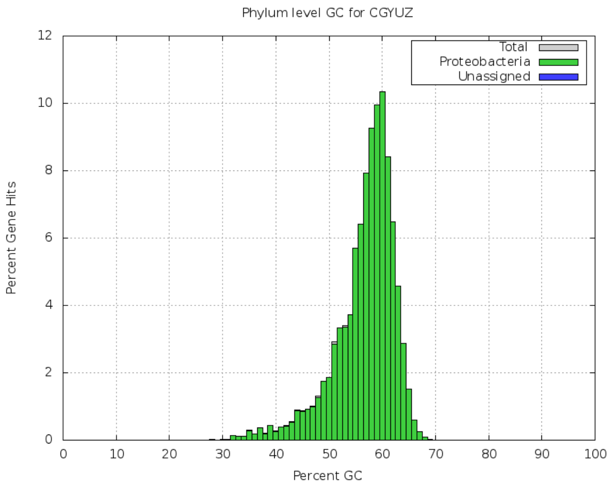

Class

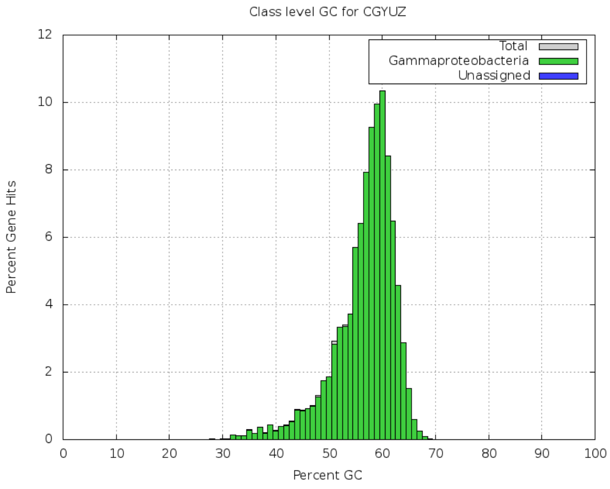

Order

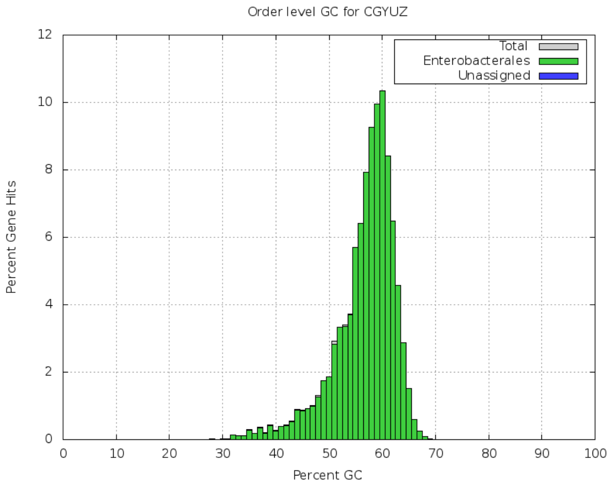

Family

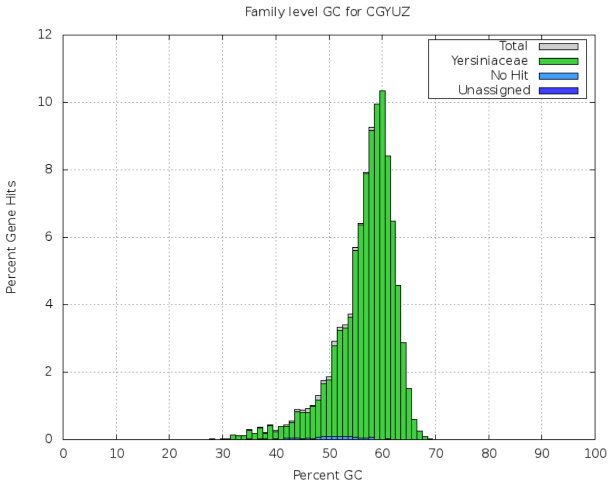

Genus

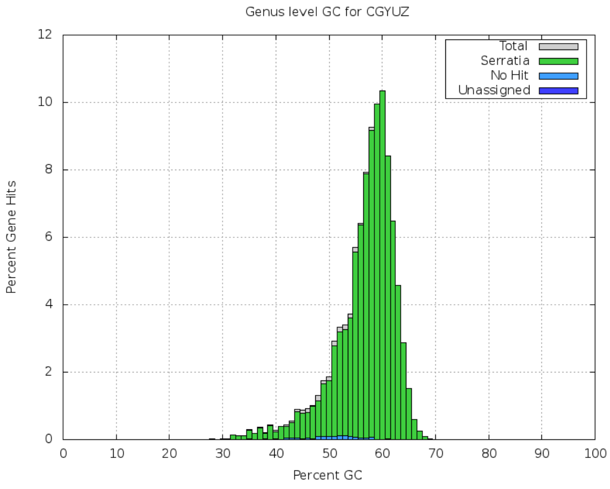

Species

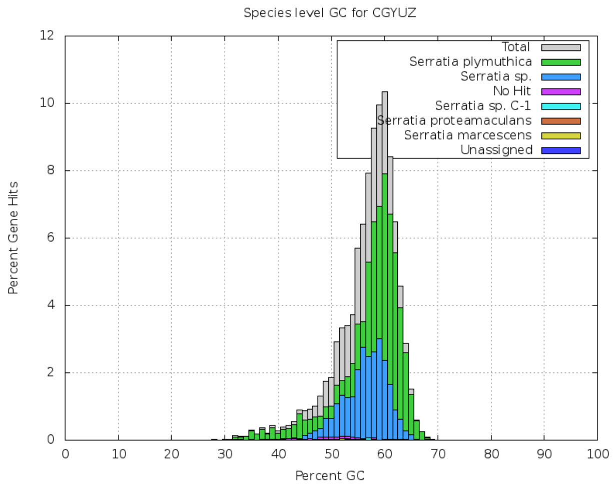

GC verses coverage of assembled scaffolds, overlaid with Silva SSU gene hits and NCBI nt megablast hits shown for different taxonomic levels.

Kingdom

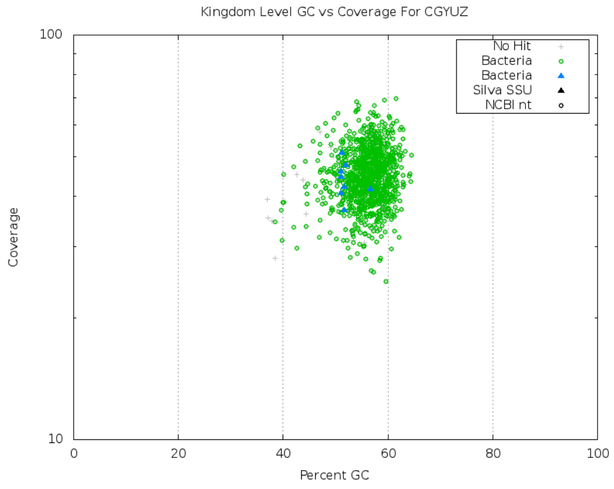

Phylum

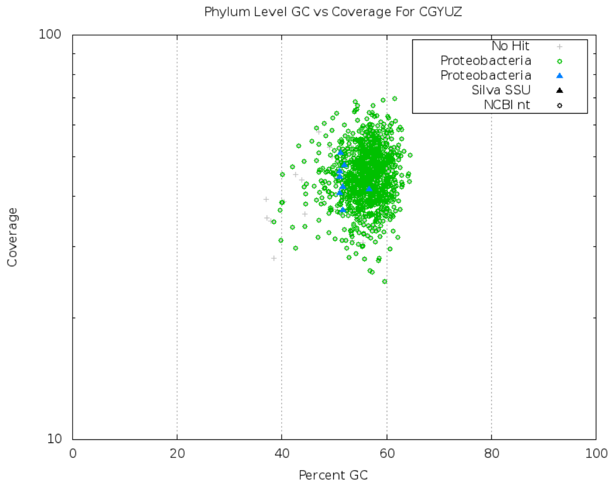

Class

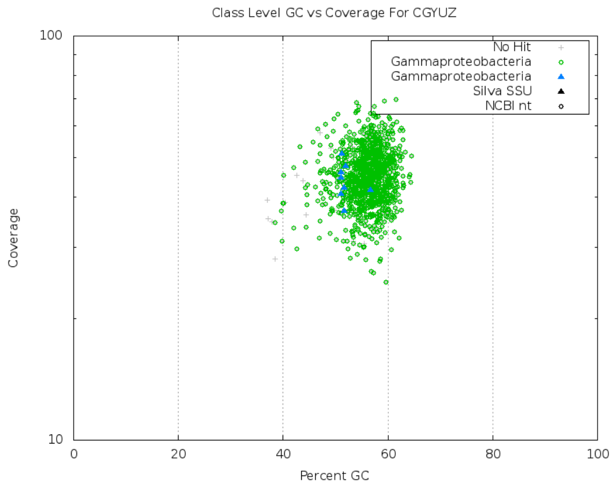

Order

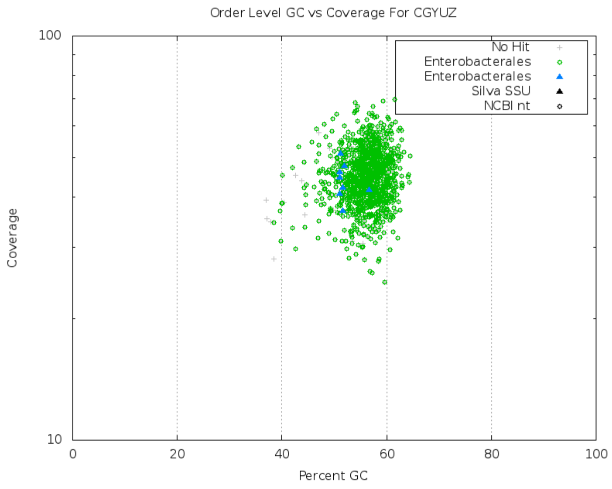

Family

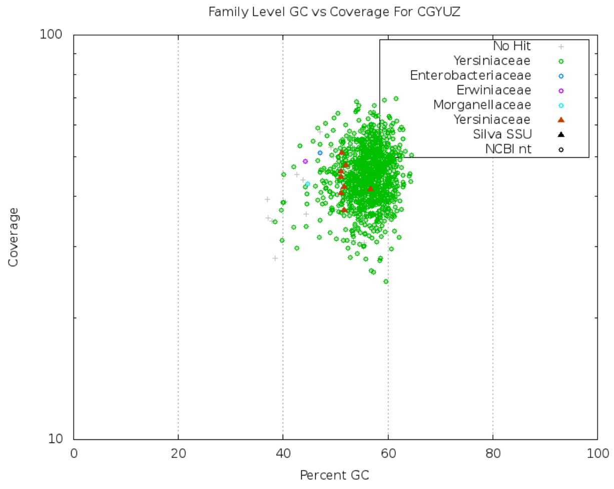

Genus

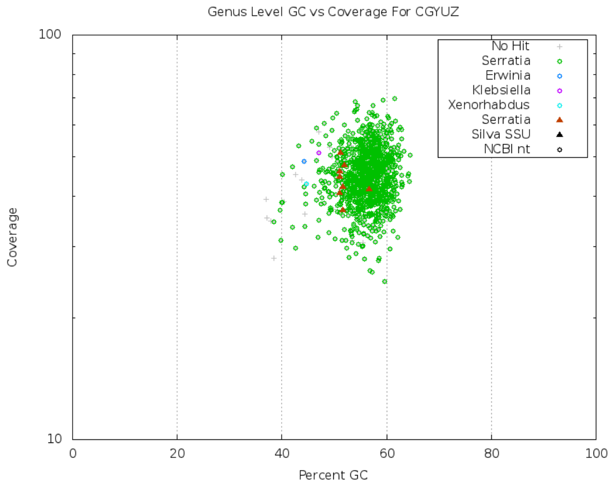

Species

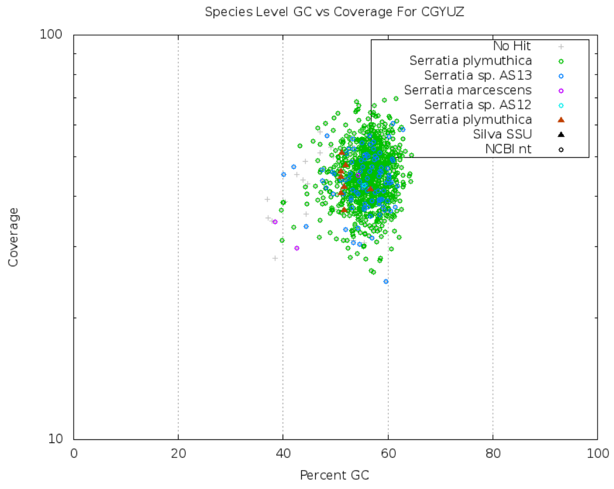

Coverage and GC information. Scaffolds were shredded into non-overlapping 5 kbp fragments and the GC content of each shred was plotted as a data point, colored by scaffold id. Coverage was calculated by mapping the fragment library to the final assembly and plotted as connected points.

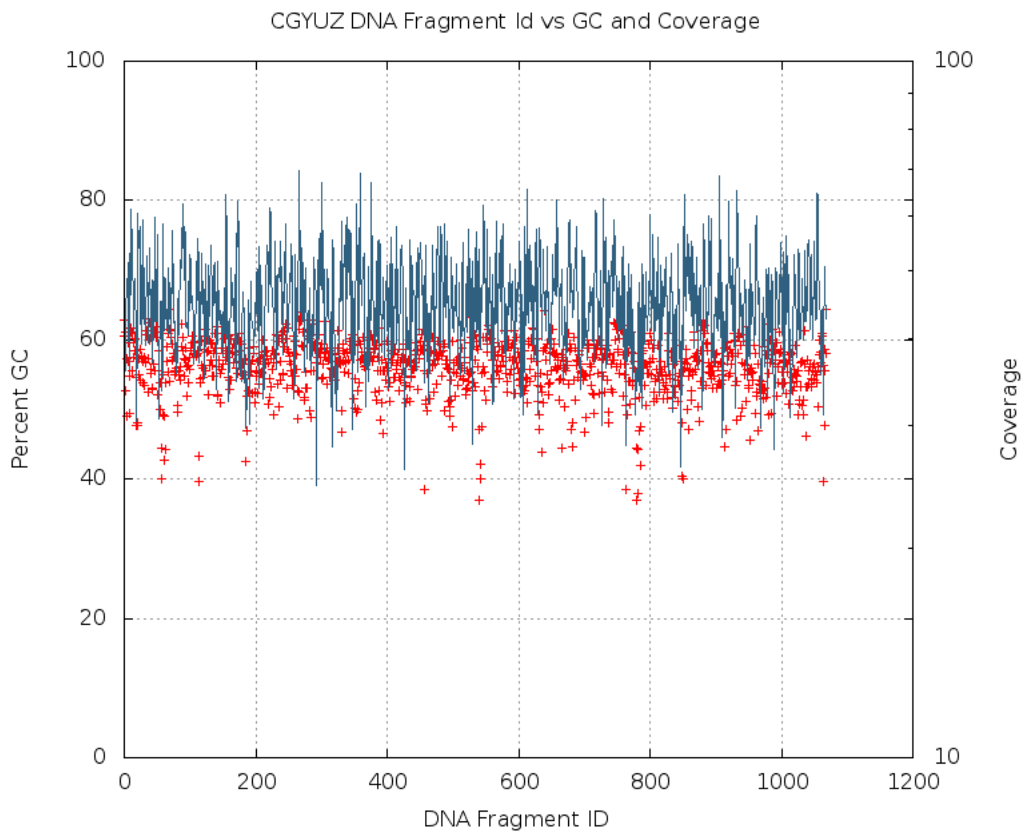

GC histogram of the scaffolds, including scaffold length weighted distribution.

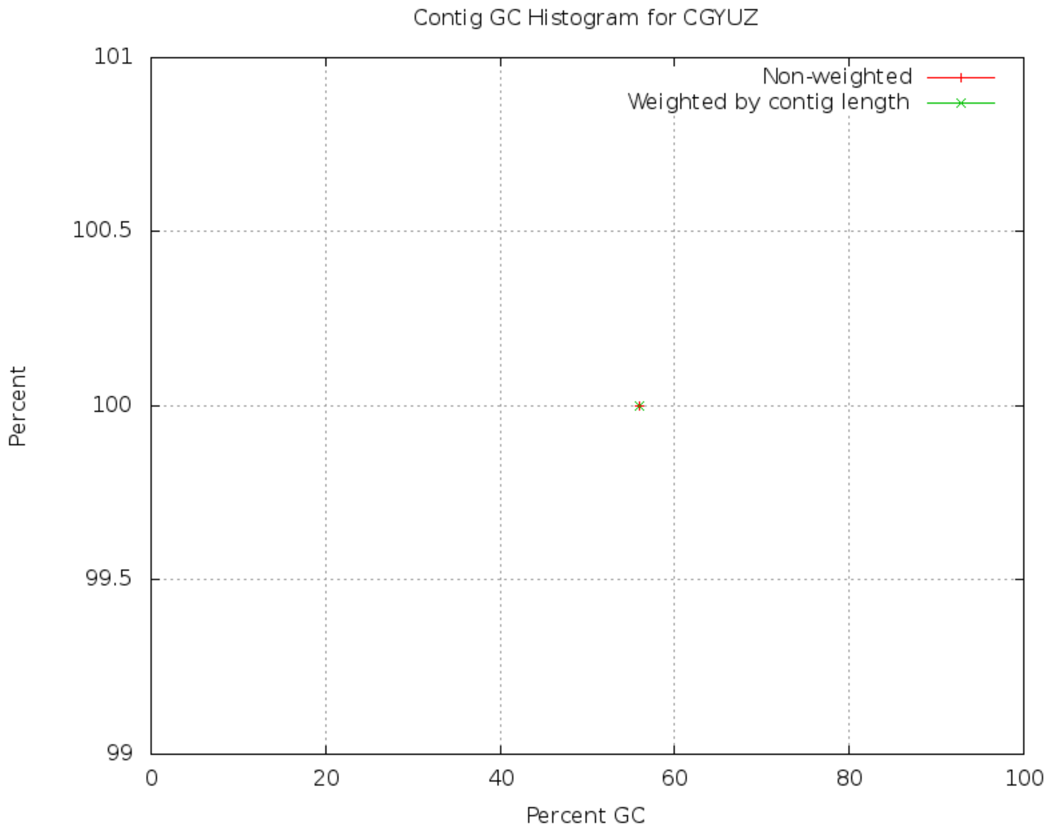

List of the top scaffold megablast hits against 16s ribosomal genes using the Silva SSU database.

Organism: N/ABacteria;Proteobacteria;Gammaproteobacteria;Enterobacterales;Yersiniaceae;Serratia;Serratia plymuthica;
Contig Name: CGYUZ_unitig_0|arrow
Align Length: 1,522 bp
Percent Id: 100.00%

Tetramer frequencies are calculated over 5kb sliding windows of all scaffolds, followed by principal component analysis. Plots of the first two principal components are colored by scaffold.

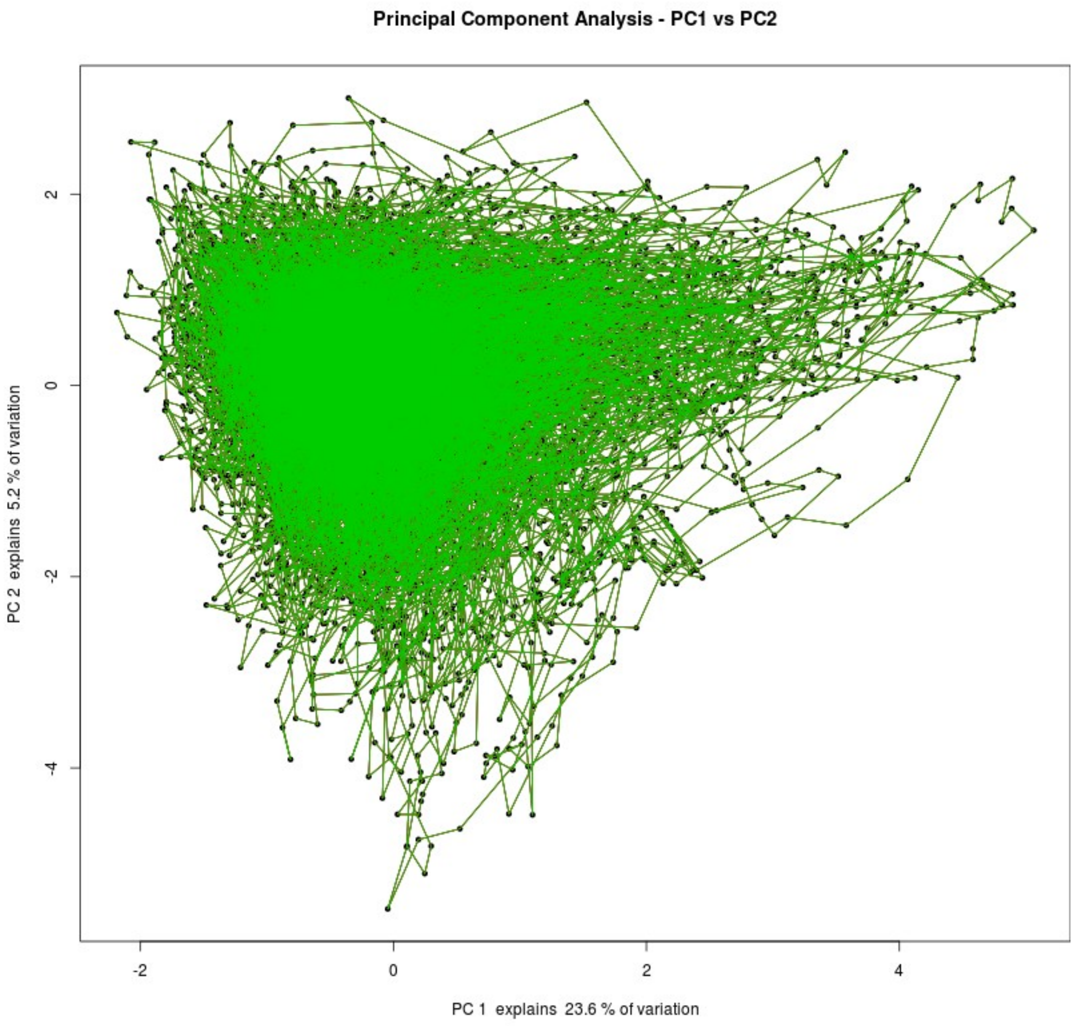

Estimated genome recovery derived from analysis of universal single-copy genes detected in final assembly using CheckM.

HMM	Found Genes	Total Genes	Percent Recovered
Archaea	70	149	46.98%
Bacteria	103	104	99.04%
Lineage Workflow	544	546	99.63%

Bacteria Single–copy Gene Histogram

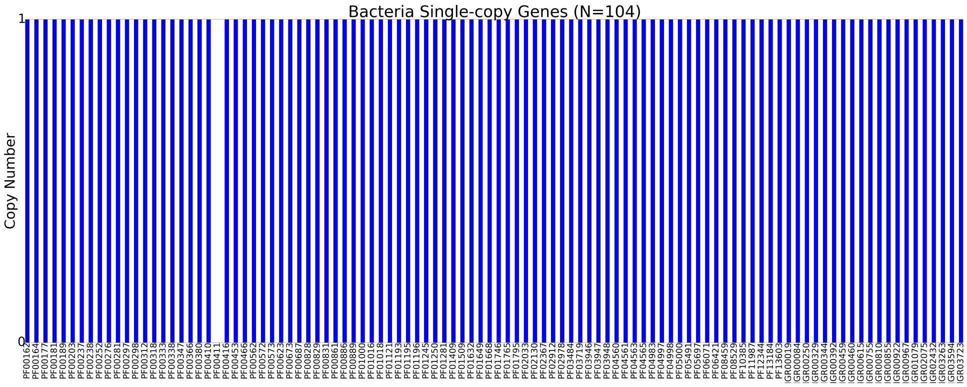

Archaea Single–copy Gene Histogram

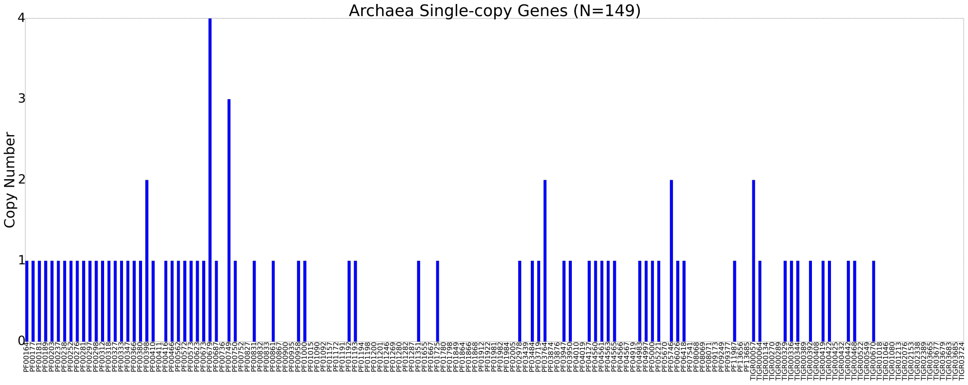

Lineage Workflow Single–copy Gene Histogram

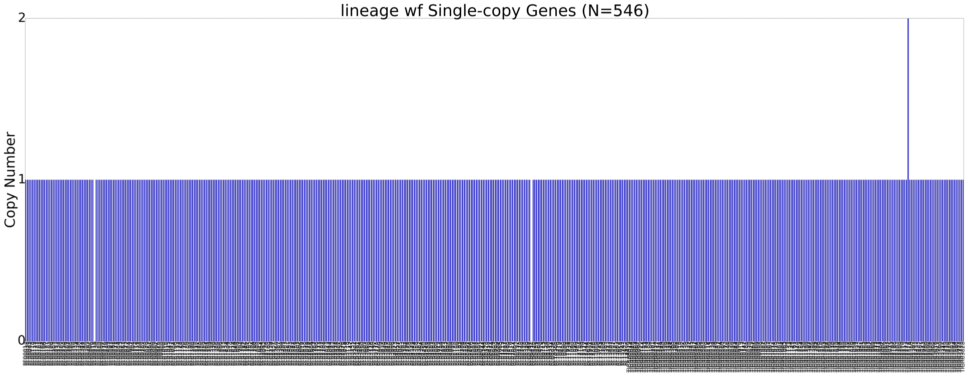

Bacteria Single–copy Genes

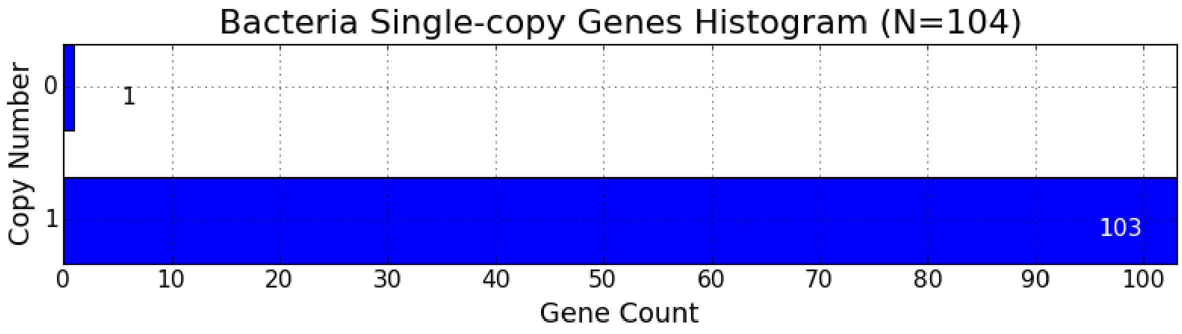

Archaea Single–copy Genes

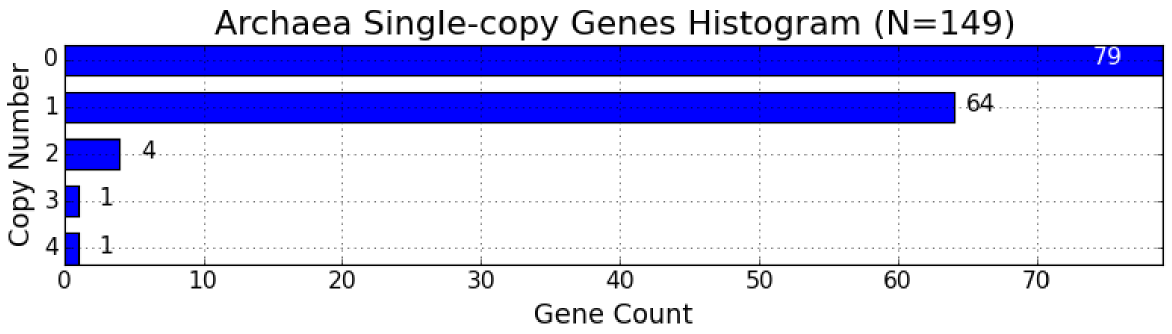

Lineage Workflow Single–copy Genes

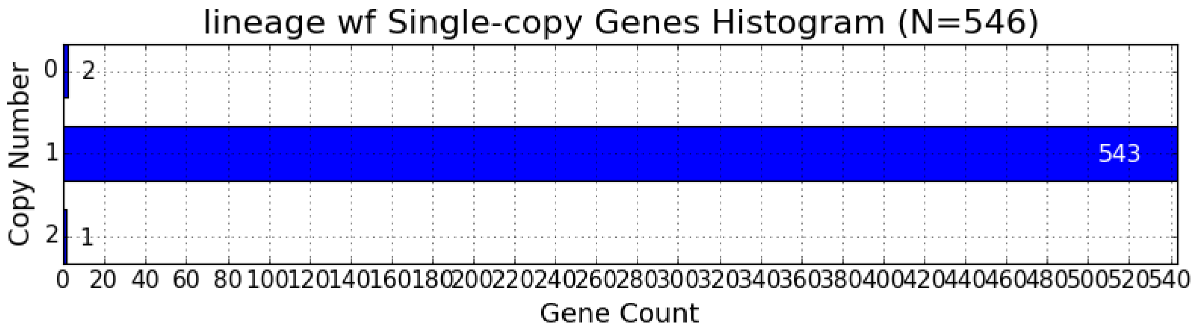

5. Sequence Data Availability

The sequence fasta files can be downloaded from our JGI portal website.
http://www.jgi.doe.gov/genome-projects

6. Methods

Isolate Improved Draft

Genome Sequencing and Assembly

The draft genome of *Serratia plymuthica* was generated at the DOE Joint Genome Institute (JGI) using the Pacific Biosciences (PacBio) sequencing technology [1]. A >10kpb Pacbio SMRTbell™ library was constructed and sequenced on the PacBio RS2 platform, which generated 78,182 filtered subreads totaling 307,584,001 bp. All general aspects of library construction and sequencing performed at the JGI can be found at http://www.jgi.doe.gov. The raw reads were assembled using HGAP (smrtanalysis/2.3.0_p5, HGAP 3) [2]. The final draft assembly contained 1 contig in 1 scaffold, totaling 5,349,225 bp in size. The input read coverage was 45.2X.

1. Eid John, et al. RealDNA Sequencing from Single Polymerase Molecules. Science 2008
2. Chin C, et al. Nonhybrid, finished microbial genome assemblies from longread SMRT sequencing data. Nat Methods 2013

DOE Auspice Statement for Publication

The work conducted by the U.S. Department of Energy Joint Genome Institute, a DOE Office of Science User Facility, is supported under Contract No. DE-AC02-05CH11231.

The data was generated for JGI Proposal #503161.
